# Cyberbullying Among Adolescent Bystanders: Role of Affective Versus Cognitive Empathy in Increasing Prosocial Cyberbystander Behavior

**DOI:** 10.3389/fpsyg.2018.00799

**Published:** 2018-05-30

**Authors:** Julia Barlińska, Anna Szuster, Mikołaj Winiewski

**Affiliations:** Faculty of Psychology, University of Warsaw, Warsaw, Poland

**Keywords:** cyberbullying, cyberbystanders, adolescents, affective empathy induction, cognitive empathy induction, prosocial cyberbystander behavior

## Abstract

The purpose of this study was to investigate if affective (vicarious sharing of emotions) and cognitive empathy (mental perspective taking) induction may stimulate adolescent online bystanders’ intervention in cyberbullying cases. The role of reporting the abuse is crucial because it is a form of active support to the victim, initiated by children, to stop the bullying. The effectiveness of empathy activation in decreasing negative cyberbystander reinforcing behavior has been proved in previous studies. The effects of affective and cognitive empathy activation on positive cyberbystander behavior, defined as reporting the bullying online, were explored in two follow-up studies *N* = 271 and *N* = 265. The influence of experiencing cyberbullying as perpetrator, victim, and as determined by gender on prosocial cyberbystander behavior was also controlled. The results indicate that only cognitive empathy activation increases the likelihood of intervening bystander behavior. Neither affective empathy induction, previous experience of cyberperpetration, cybervictimization, nor gender affected the engagement in prosocial bystander behavior. The conclusion of the research is that a program consequently activating more reflective cognitive empathy induction can contribute toward the establishment of healthier behavioral patterns among bystanders to cyberbullying, increasing the probability of their reporting the cyberbullying acts.

## Introduction

One of the most serious threats to individual and social well-being online is cyberbullying among adolescent internet users. It is an extremely damaging type of interpersonal violence present in schools throughout different countries (Kowalski et al., 2014; Zych et al., 2015; Smith et al., 2016; Wright et al., 2016). In most cases, cyberbullying is interconnected with school bullying and has an important negative impact on aggressive behavior at school and mental health outcomes (Beran and Li, 2007; Juvonen and Gross, 2008; Pyżalski, 2013; Fletcher et al., 2014). Cyberbullying engages a wide scope of groups and roles among pupils – victims, perpetrators and witnesses. Given this broad impact, it often becomes a problem for the entire school culture and often beyond – a social problem. Previous research has shown that cyberbullying can be more serious (as perceived by the victims) than traditional bullying, mainly due to the (often inevitable) wide publicity of online attacks (Smith et al., 2008; Sticca et al., 2013). It thus has the potential for an almost unlimited audience. The challenge of escaping or controlling the harassment, focusing on exploring ways to increase helpful responses to online harm, seems a crucial task. Empathy plays a central role in human behavior (Hogan, 1969) also in the online context (Barlińska et al., 2013). Thus it seems essential in regulating the prosocial behaviors of bystanders to cyberbullying.

### Cyberbullying

Nowadays cyberbullying has become a common occurrence and a substantial concern. The way the phenomenon is defined has an impact on prevention and intervention practice. One of the most commonly used definitions is “any behavior performed through electronic or digital media by individuals or groups that repeatedly communicates hostile or aggressive messages intended to inflict harm or discomfort on others” (Tokunaga, 2010; p. 278). Some researchers emphasize cyberbullying’s similarity to traditional bullying (Olweus, 2012). Others highlight the need for a different understanding, questioning the adequacy of the classic criteria of peer violence as it relates to cyberbullying (Menesini et al., 2012; Palladino et al., 2017).

Peers, social status, and student–teacher relationships play a dominant role in the socialization of adolescence, both online and offline (Hinduja and Patchin, 2013; Longobardi et al., 2018). This highlights the importance of bystanders as a powerful social influence in creating positive anti-bullying behavioral models, with such responses as intervention in cyberbullying cases (Salmivalli et al., 1996; Menesini et al., 2003; DeSmet et al., 2012; Barlińska et al., 2013, 2015; Macháčková et al., 2013, 2015; Bastiaensens et al., 2014; Pfetsch, 2016). Research into cyberbullying has recently turned its attention to the role of cyberbystanders. It has been found that, across studies, prevalence rates of cyberbystanders vary just as in cases of cyberperpetration and cybervictimization, possibly due to different methodological approaches (e.g., formulation of questions, reference time frames or cut-off criteria), age ranges, or cultural differences (Cook et al., 2010). In total, prevalence rates for cyberbystanders range between 20 and 55% (Pfetsch, 2016); these are higher rates than for mean prevalence rates of around 15% for cyberbullies and cybervictims (Modecki et al., 2014). Such high scores justify the need for prevention programs focused on the role of bystanders. Encouraging cyberdefenders’ tendency to report online bullying is crucial, as it is one of the most effective ways to support the victim(s) (O’Neill and McLaughlin, 2010; Livingstone et al., 2011).

In general, researchers differentiate several typologies of bystanders in both the offline and online contexts (Salmivalli et al., 1996; Olweus, 2001; DeSmet et al., 2012; Bastiaensens et al., 2014; Shultz et al., 2014). While there are some differences, what is underlined in each model is the importance of prosocial bystander behavior as an effective solution to bullying. The data prove that the picture of cyberbystanders’ reactions is more complex than in traditional bullying, mainly due to the specifics of computer-mediated communication (DeSmet et al., 2012, 2016; Barlińska et al., 2013, 2015; Macháčková et al., 2013, 2015; Bastiaensens et al., 2014; Obermaier et al., 2014; Pfetsch, 2016). Given the reduced social and contextual cues available (Kiesler et al., 1984), chances of prosocial reactions to the cyberbullying acts are lessened.

In terms of modifying cyberbystanders’ participation in online bullying, the few studies in this area have demonstrated the importance of emphasizing a triadic approach and focusing on group processes, as a means of fully understanding and effectively moderating the phenomenon of cyberbullying (Barlińska et al., 2013, 2015; Macháčková et al., 2015; Pfetsch, 2016).

A number of intervention programs designed to tackle cyberbullying have already been developed (i.e., Menesini et al., 2012; Williford et al., 2013). Some even exclusively target cyberbullying (e.g., Ortega-Ruiz et al., 2012; Schultze-Krumbholz et al., 2016), and some include bystander or peer support elements, as have proved effective in reducing victimization from cyberbullying on the global level (Salmivalli et al., 2011; Menesini et al., 2012; Palladino et al., 2012). Nevertheless, one area that remains largely unknown involves which specific factors can encourage bystanders’ intervention in cyberbullying. Studies on cyberbystanders showed that, as in offline bullying, most bystanders witnessed passively and took no action (Salmivalli, 2010; Dillon and Bushman, 2015; Song and Oh, 2018). These data legitimate the need for exploring how to effectively increase bystander intervention in cyberbullying – both in terms of effective factors and establishing a successful ethos of activating such factors.

In the current investigation, targeting positive bystander behavior (understood as reporting the harassing act) was chosen as a viable approach to reduce cyberbullying. One of the most natural factors reducing various forms of aggression and having the potential to influence cyberbystanders’ prosocial reactions is empathy.

### Empathy: The Affective and Cognitive Aspects

Empathy is described as an affective response that is more appropriate to the situation of the other person than to one’s own (De Vignemont and Singer, 2006). This is a complex and multidimensional phenomenon that includes, on the one hand, the ability to notice, feel, and automatically respond to other people and, on the other, to understand their emotional states (Batson, 1991; Hoffman, 2000). Empathy has been often associated with prosocial behavior as the crucial condition of sharing and understanding the emotional and mental states of others (Eisenberg and Strayer, 1987). The term empathy is used to refer to two related, yet different human abilities: mental perspective taking (cognitive empathy) and the vicarious sharing of emotion (affective empathy) (Batson, 1991; Davis, 1996; Hoffman, 2000).

Affective empathy reflects the innate, automatic capacity to respond with arousal to the signs of discomfort or other affective states of the other. It is initiated through direct contact, which ensures access to species-universal information that activates affective empathy mechanisms (Preston and De Waal, 2002). The empathic arousal mechanisms, which appear at an early stage of human development, are the circular reaction and motor imitation. At a later stage, responses to another person’s circumstances become increasingly governed by cognitive factors based on learning mechanisms. This is how cognitive empathy develops (Hoffman, 2008).

Cognitive empathy, defined as the ability to understand the beliefs, feelings and intentions of the other (Decety and Jackson, 2004; Decety, 2007) involves more complex cognitive processes and empathy triggering mechanisms (Hoffman, 2000). Through classical conditioning and association, other people’s perceived emotions can be related to our own past experiences. Due to language-mediated association, empathic arousal becomes independent from the present or past contact with another person. The most advanced mechanisms, i.e., understanding various roles and perspective taking, allow us to anticipate the consequences of our actions for other people. Contrary to genetically determined affective empathy (Matthews et al., 1981; Rushton et al., 1986; Zahn-Waxler et al., 1992), cognitive empathy is driven primarily by environmental factors, such as parental or school influence (Baron-Cohen, 2011). Modeling, inducing, and perspective-taking are often mentioned as parenting techniques facilitating the development of cognitive empathy (Hoffman, 2000). They are also basic techniques implemented in school programs.

The most frequently mentioned aspects of empathy are its social significance and benefits associated with morality, altruism, fairness, prosocial and helping behavior, and cooperativeness (Eisenberg and Miller, 1987; Batson and Shaw, 1991; Eisenberg and Morris, 2001; Hoffman, 2008). Empathy is indicated as one of the mechanisms of prosocial behavior or altruism (i.e., empathy–altruism hypothesis; Batson et al., 1987; Batson, 1991, 2011; Hoffman, 2000, 2008; Szuster, 2016).

Empathy activation strategies have been included in intervention programs (Chandler, 1973; Chalmers and Townsend, 1990), where they have proved effective in promoting prosocial bystander behaviors and reducing both offline and online bullying in schools (Salmivalli et al., 2011).

Empathy is also correlated positively with emotion regulation and emotional behavior (Eisenberg, 2000), and negatively with negative emotions frequency (Davis et al., 1996). It is also connected with a sense of guilt, which in turn can stimulate prosocial behavior (Hoffman, 2008). This kind of activation may be found to effectively increase prosocial behavior also in cyberspace.

Less is known about which kind of empathy (affective or cognitive) is more effective in inducing helping behavior. Although numerous studies confirm the connection between both types of empathy and help offering (Davis, 1996; Eisenberg, 2000; Batson, 2011; Bloom, 2016), the nature of such help is different depending on whether it is motivated by affective or cognitive empathy. Since the beginning of scientific interest in the phenomenon, cognitive empathy was found to be the underpinning of long-term cooperation (Smith, 1759/1976). Contemporary research findings show links between cognitive empathy and anticipation of long-term consequences of help-giving (Batson and Ahmad, 2001), limiting victim blaming (Lerner, 1977), as well as modifying the established stereotypes related to potential help benefactors (Batson and Ahmad, 2009). On the other hand, affective empathy inducing universal mechanisms not only provides a buffer against aggression, but is also the first and foremost mechanism generating helping behaviors (Piliavin et al., 1982). This profound effect of affective empathy, manifested in the form of numerous donations for people in need whose images are created through media, can easily be observed in hundreds of social campaigns (Bloom, 2016).

Experimental evidence confirming the relation between empathy and altruism (Batson, 1991) indicates that cognitive empathy (corresponding with empathic care and focused on understanding the emotional states of others) is conducive to help-offering, irrespective of situational factors (such as mood or how easily helping may be avoided), whereas help yielded by affective empathy (corresponding with emphatic anger) is of a conditional character. It is generated when there is no other way to reduce the discomfort caused by the suffering of another person.

This consistent concept indicating a regulatory role of empathy in social functioning remains the subject of diverse discussions (Bloom, 2016; Jordan et al., 2016). Illustrative of these differences is the contrast between feeling what you believe others feel (often described as empathy) and caring about the welfare of others (often described as compassion or concern). Research which explored the relationship between the Empathy Index and measures of concern and cooperative, altruistic behavior revealed that empathy and concern consistently load on different factors (Jordan et al., 2016). Furthermore, it showed that empathy and concern motivate different behaviors: concern for others is a uniquely positive predictor of prosocial action, whereas empathy is either not predictive or negatively predictive of prosocial actions. This limits a monolithic, mostly positive character of regulatory effects commonly identified with empathy and provokes a more selective way of thinking about the very nature of the phenomenon. Nevertheless, the fundamental question: what is the empathy constituting factor – still remains valid, as the mechanism operates on various levels and is dependent upon a special brain circuit that consists of as many as 12 cerebral centers (Stone et al., 1998; Lamm et al., 2007; Shamy-Tsoory et al., 2009).

The above data give rise to a question regarding the role of the two types of empathy in regulating the prosocial behaviors of bystanders to cyberbullying.

### Empathy and Cyberbystanders

The regulatory role of empathy in bystanders’ reactions to cyberbullying has been demonstrated by the results of various studies (Barlińska et al., 2013, 2015; Pfetsch and Ittel, 2014; Macháčková et al., 2015; Pfetsch, 2017). Empathy has consistently been found to predict defending victims of both traditional bullying (Nickerson et al., 2008) and cyberbullying (Macaula and Boulton, 2017). Data focused on cyberbystanding point to empathy as one possible protective factor against negative online behavior (as a cyberbully or passive cyberbystander) (Barlińska et al., 2013, 2015; DeSmet et al., 2016), and also as one that increases the probability of prosocial online behavior (supporting the victim) (Pfetsch and Ittel, 2014; Macháčková et al., 2015; Macaula and Boulton, 2017). Several studies show that persons with higher dispositional empathy may be more likely to intervene in a prosocial manner (Freis and Gurung, 2013; Macháčková et al., 2013; Macaula and Boulton, 2017).

Additionally, researchers show that activation of empathic reactions appears to be dependent upon situational factors related to cyberbullying incidents such as specific technological settings, friend vs. acquaintance of the cybervictim, the bully’s popularity, clear vs. unclear circumstances, perceived fairness of the behavior of involved parties, directness or proximity of the cyberbystander to the cybervictim, severity of the act, receiving a request for help from the victim or not, etc. (DeSmet et al., 2014; Macháčková et al., 2015, 2016; Palladino et al., 2017). This justifies the growing need to explore the effectiveness of situational empathy induction as a factor potentially increasing cyberbystander interventions. Such findings may provide a basis for launching school and evidence-based anti-cyberbullying education projects.

In our previous studies on empathy activation with respect to cyberbystander behavior (Barlińska et al., 2013, 2015; Szuster et al., 2016), the effectiveness of empathy in decreasing cyberbystander reinforcing behavior has been proved. However, to date only the potential to diminish the scale of cyberbullying via empathy activation using this method has been applied; thus, further exploration is needed.

### The Role of Cyberperpetration and Cybervictimization Experience

Substantial evidence clearly points to various links between offline and online bullying roles in terms of: cyberperpetration as a predictor of traditional school bullying (Raskauskas and Stoltz, 2007; Ybarra and Mitchell, 2007; Juvonen and Gross, 2008; Dehue et al., 2012; Sticca et al., 2013); traditional school bullying perpetration experience as a cyberperpetration predictor (Ybarra et al., 2007; Dehue et al., 2008; Erdur-Baker, 2010; Twyman et al., 2010); and links between the roles (the bully–victim status) (Ybarra and Mitchell, 2004; Walrave and Heirman, 2011; Dehue et al., 2012). The role overlap between cyberbystanders, cyberbullies, and cybervictims offers evidence that roles in cyberbullying acts are not mutually exclusive (Pfetsch and Ittel, 2014).

The limited research that relates to predicting cyberbystander behavior from previous experience of cybervictimization and cyberperpetration presents important findings. Being a cyberbully has proved to be an important predictor of reinforcing cyberbystander behavior (Fawzi and Goodwin, 2011; Barlińska et al., 2013, 2015; Szuster et al., 2016). On the other hand, helping the victim was predicted by victimization in both traditional bullying and cyberbullying (Fawzi and Goodwin, 2011), though some studies (Barlińska et al., 2013, 2015) showed no impact.

The relationship between cyberperpetration and cybervictimization experience and cyberbystanders’ intervening behavior has also been verified in the present study.

### Cyberbystander Behavior and Gender

Gender is a variable traditionally present in the exploration of aggressive behavior both in face-to-face and online contexts.

Previous research on gender differences regarding cyberbullying incidents has provided inconclusive findings. On the one hand, two meta-analyses (Cook et al., 2010; Barlett and Coyne, 2014) showed a significantly higher involvement of boys in cyberperpetration; further, girls more often than boys fall victim to cyberviolence. However, these differences were found to be rather negligible. On the other hand, other studies have found that girls are more likely than boys to be cyberbullies (Pornari and Wood, 2010), especially in more indirect forms of online aggression, such as rumor-spreading through Internet blogs and circulation of photos/videos. Alternatively, a systematic narrative review conducted by Tokunaga (2010) revealed that most of the studies showed no gender differences with respect to cyberperpetration or cybervictimization rates.

In many aspects gender differences in cyberbystander reactions remain even more equivocal. In some studies females were found to offer greater support and assistance than males when witnessing cyberbullying, and were more often nominated as peer helpers (Rigby and Slee, 1991; Menesini et al., 1997, 2003; Oh and Hazler, 2009; Bastiaensens et al., 2014; Quirk and Campbell, 2014); in other research no gender differences were found in both positive and negative bystander reactions to cyberbullying (Li, 2006; Fawzi and Goodwin, 2011; Barlińska et al., 2013, 2015; Macháčková et al., 2013; Szuster et al., 2016).

The relationship between gender and the cyberbystanders’ intervening behavior has also been controlled in the present study.

### Current Research

The role of reporting cyberabuse is crucial, for two reasons. First, it is a form of active support to the victim initiated by children to stop the bullying (Pfetsch, 2016; Smith, 2016). Second, it is the only form of support with the potential of activating an intergenerational and multi-shareholder reaction to bullying (Livingstone et al., 2011).

The main objective of the current research was to explore the effectiveness of affective and cognitive empathy activation in stimulating adolescents’ intervention in cyberbullying cases. The effectiveness of empathy activation in decreasing negative cyberbystander reinforcing behavior has been proved in our previous studies (Barlińska et al., 2013, 2015; Szuster et al., 2016). This induction, tested on Polish junior high school students (*N* = 2411), was found to significantly and repeatedly reduce the reinforcing cyberbullying response. In the current follow-up series of two studies, a verification was conducted to determine whether empathy inductions may be a viable option for stimulating cyberbystanders to react prosocially and report cyberbullying abuse.

Two other factors which have proved to modify adolescent reactions toward cyberbullying were also included – cyberperpetration and cybervictimization experience and gender. Previous viewing of the material used to activate empathy was also controlled for.

It was expected that activation of both affective and cognitive empathy would increase frequency of behaviors aimed at helping cyberbullying victims. Affective and cognitive empathy were activated in two separate experimental studies. It was anticipated that higher odds would be found of choosing prosocial cyberbystander behavior understood as active reporting of online bullying in the experimental groups (where empathy was inducted), compared to control groups (without any induction). The influence of experience of cyberbullying as perpetrator, victim, and the role of gender on positive bystander behavior was also controlled.

## Study 1

As argued before, affective empathy preceding a cyberbullying act may increase the probability of cyberbystander helpful reactions throughout automatic activation of emphatic arousal. Study 1 was designed to test whether affective empathy activation is associated with a higher likelihood of cyberbystander intervening behavior. Activation of affective empathy preceding a potential cyberbullying act may increase the probability of cyberbystander helpful responses through automatic activation of emphatic arousal. We decided to test whether affective empathy activation is associated with a higher likelihood of cyberbystander intervening behavior.

### Method

This experimental study was conducted using a web application that simulated a social networking site and a messaging service. The study was approved by the ethics committee of the faculty of Psychology of the University of Warsaw.

### Participants

Participants were junior high school students (*N* = 271, comprising 121 boys and 151 girls) from 10 public junior high schools located in an average socioeconomic status neighborhood in three Polish districts. All students were between the ages of 11 and 17 years (*M*_age_ = 13.05 years, *SD*_age_ = 0.80). The selection for the sample was purposeful and was carried out in cooperation with the Polish Saferinternet awernode^1^, who sent invitations for participation in the study to schools reporting problems with cyberbullying. Assignment to the experimental or control conditions was done by drawing halves of the classes.

### Procedure

The study was anonymous and conducted in groups and on school premises; written informed consent was obtained from the headmaster, parents, and pupils. Students were randomly assigned to control and experimental groups. Each participant logged in using a unique, one-time password that provided access to study material. The research took place at the school during computer classes. The full study was preceded by a technical pilot with the participation of teachers on computers in IT laboratories where the research was carried out. The duration of the procedure was about 20 min. Students who did not take part in the study were offered an alternative educational activity.

The first task was different for the experimental vs. control group. In the experimental group participants watched a 2-min film (the story of a victim of cyberbullying), while the control group viewed no exposition. Then, the participants in all groups received the second task, “Message from a friend”. After reading the message, they were asked to choose how to act: report vs. send (see **Table 1**). Next, they completed a questionnaire of the experience of cyberbullying victimization and perpetration. Finally, questions on age, province of the school, and gender were asked. At the end of the study, students, teachers, and parents were provided with general feedback.

**Table 1:** Study design I – conditions and indicators.

Study conditions	Behavior of a cyberbystander	
	Cyberbullying intervening behavior	Cyberbullying reinforcing behavior
Activation of affective empathy (specific for a given situation)	Report the abuse	Send
Control group		

### Measures

#### “Film”

To activate affective empathy, a 2-min video recording was used presenting a case of cyberbullying, the victim’s feelings, and the effects on her behavior. It told the story of a young girl who became a victim of cyberbullying by being filmed by a school colleague while she was dressing for physical education in a locker-room. The video was posted on the web and gradually gained popularity; as a result, she became a figure of derision. She experienced strong negative feelings and emotions such as shame, humiliation, ridicule and fear. This situation also had an impact on behavioral expressions: isolation from contemporaries, staying at home, and school absenteeism. The film showed the course of events and consequences by presenting the cyberbullying victim’s behavior. It also included a statement expressing the girl’s feelings and experiences. The procedure and its effectiveness in empathy activation^2^ has been validated and applied in several similar studies (Barlińska et al., 2013, 2015; Szuster et al., 2016). As the film is already implemented in an anti-cyberbullying school program, we additionally asked participants whether they had seen the film before (for later use as a control variable in the model).

#### “Message From a Friend”

To simulate social youth interactions in cyberbullying situations, a special application called “Message from a friend” was used. At the beginning of this simulated peer interaction, pupils are having a short chat with a virtual friend who, at the end of the chat, sends a message insulting a different pupil (a photomontage presenting a dog with a boy’s head) with the following comment: “Hi, this is my classmate, he looks like a total fool.” The situation was inspired by cases reported to the Polish helpline.org.pl website (part of the Safer Internet project that provides support to the victims of Internet threats). The participants could choose between sending the insulting message forward (cyberbullying reinforcing behavior) or reporting it (positive intervening behavior).

#### Cyberbullying Questionnaire

A questionnaire about cyberbullying experience (Barlinìska and Wojtasik, 2008) was employed. The questionnaire consists of two parts, each containing 10 questions related to the experience from the perpetrator’s perspective (e.g., “Have you ever posted or sent material that was false or embarrassed someone?”) and that of the victim (e.g., “Has anyone ever posted false or embarrassing materials about you?”). Answers are indicated on a 4-point Likert-type scale (1 – never, 4 – several times). Both scales (*M*_victim_ = 0.40, *SD* = 0.52, and *M*_perpetrator_ = 0.38 *SD* = 0.52) proved to be internally consistent, α = 0.73 and α = 0.77, respectively. The composite scores were used in further analyses.

### Plan of Analysis

All analysis were conducted using SPSS 25. The logistic regression model was chosen due to having a dichotomous dependent measure and several continuous and binary predictors. It is reported following Peng, Lee, Ingersoll guidelines (Peng et al., 2002). Analysis was conducted to evaluate whether the activation of affective empathy rose to the likelihood of intervening cyberbystander behavior. Additionally, it was considered whether, as in prior research findings (Barlińska et al., 2013, 2015; Szuster et al., 2016), the experiences of cyberperpetration influenced the frequency of choosing helping behavior. The impact of gender and cybervictimization on cyberbystander behavior, which was not significant in previous studies (Barlińska et al., 2013, 2015; Szuster et al., 2016) was controlled. Additionally, previous viewing of the material was controlled.

### Results

To assess the impact of affective empathy on cyberbystander intervening behavior, we conducted a logistic regression analysis (**Table 2**). The analysis showed that the model was not significant. There were no missing values and all analyses were conducted on the full sample. All the overall model statistics turned out to be suboptimal. Neither manipulation of affective empathy nor any of the controls (cyberbullying history, age, or gender) turned out to be significant in predicting helping bystander behavior.

**Table 2:** The results of the logistic regression analysis for activation of affective empathy, cyberperpetration, cybervictimization, gender, and previous viewing of the film on intervening cyberbystander behavior.

Predictor	*B*	*SE B*	Wald’s χ^2^	OR [CI95%]
Control film (0 – didn’t see)	-0.29	0.41	0.50	0.75 [0.33–1.67]
Gender (0 – boys)	0.10	0.26	0.14	1.10 [0.66–1.84]
Cyberperpetration	0.09	0.31	0.07	1.09 [0.59–2.01]
Cybervictimization	-0.34	0.31	1.22	0.71 [0.38–1.30]
Affective empathy (0 – no empathy)	0.39	0.26	2.20	1.47 [0.88–2.46]

**Overall model**			**χ^2^**	

Likelihood ratio test			2.38	
Score test			3.83	
HandL			3.33	

*Cox and Snell *R*^*2*^= 0.01; Nagelkerke *R*^*2*^= 0.02.*

### Discussion

The results of the current study indicated that affective empathy activation did not increase cyberbystander intervening behavior. In contrast, previous studies using this method (Barlińska et al., 2013, 2015; Szuster et al., 2016) revealed its potential in limiting cyberbystander reinforcing bullying behavior. The results suggested that gender does not affect cyberbystanders’ behavior, which is consistent with some of the results of other research (Li, 2006; Fawzi and Goodwin, 2011; Macháčková et al., 2013; Barlińska et al., 2013, 2015; Szuster et al., 2016). The results on lack of impact of cybervictimization on cyberbystander behavior are in line with some results on pro-bullying reactions (Barlińska et al., 2013, 2015; Szuster et al., 2016), but differ from some studies focused on tendencies to help the victim (Fawzi and Goodwin, 2011). These differences may be due to methodological differences in the measurement of cyberbystander behavior (i.e., self-reports of experiences vs. experimental manipulation); they therefore need further exploration.

The obtained results show that the regulatory role of empathy in increasing intervention in cyberbullying may be more complex than in cases of inhibiting negative and antisocial cyberbystander behaviors. For the first, the specifics of cyberspace generate limitations. Affective empathy stimulation may be more difficult in this context where, in comparison to face to face contact, emotional signals are largely unavailable (Kiesler et al., 1984). Direct contact has been demonstrated to be an important condition of the automatic nature of affective empathy activation mechanisms, whereas cognitive empathy is free from such constraints (Hoffman, 2000).

Secondly, assessing the status of affective empathy in the context of cyberbullying intervention programs is not simple. An evaluation study of a German program, “Media Heroes” (Schultze-Krumbholz et al., 2016), revealed significant effects on affective empathy only in the case of a longer intervention. The short intervention, as in the current study, did not have any effects on cyberbullying rates. This result confirms that the conditional nature of involvement in cyberbullying and its dependency upon situational factors tends to be related especially to affective empathy and cyberbystander behavior (Pfetsch, 2016). The large audience on the Internet, combined with the distance between actors, can have implications for cyberbystanders’ reactions. This is especially applicable to conditions of activation of affective empathy (Latané and Darley, 1970), leading to online passivity in intervening behavior (Macháčková et al., 2015; Song and Oh, 2018). It may be that situational activation of affective empathic responses may be not sufficient to increase the probability of prosocial online behavior (i.e., cyberbystander intervention). This would imply a greater effectiveness of cognitive empathy as a mechanism for increasing the adolescent’s prosocial online behavior.

## Study 2

The effectiveness of cognitive empathy activation in increasing the likelihood of cyberbystander intervening behavior was tested in this second study.

### Method

The same web application as in Study 1 was used. Independent variables were the activation of cognitive empathy, experience as a cyberbully, experience as a cyberbullying victim and gender. Some additional controls were introduced: the number of attempts in giving correct answers in the experimental task and previous viewing of the film. The dependent variable was a cyberbystander’s choice between intervention in cyberbullying cases (reporting the bullying act) and reinforcing cyberbullying behavior (sharing it with peers). The study was approved by the ethics committee of the faculty of Psychology of the University of Warsaw.

### Participants

Participants were junior high school students (*N* = 265, comprising 168 girls and 96 boys) of nine public junior high schools located in an average socioeconomic status neighborhood in three Polish districts. All students were between the ages of 10 and 16 (*M*_age_ = 14.14 years, *SD*_age_ = 1.65). Selection of the participant group and assignment to the experimental or control conditions was the same as in Study 1.

### Procedure

The study followed a between-participants design. The place of the investigation, procedure, feedback and consent rules were similar to those in Study 1. First, pupils were randomly assigned to experimental (empathy activation) or control (neutral activation) conditions. Next, the “Message from a friend” task, with the selection of type of behavior, was conducted. Finally, the experience of cyberbullying questionnaire was administered.

### Measures

As mentioned, the same two measures were employed as in Study 1: the application “Message from a friend” and the 10-item questionnaire of cyberbullying experience. Both instruments proved to be reliable: *M*_victim_ = 0.40, *SD* = 0.48, α = 0.61, and *M*_perpetrator_ = 0.42, *SD* = 0.58, α = 0.78.

#### “Empathy Activating Task”

The opening task in the second study was the cognitive empathy manipulation. Its effectiveness was previously established (Barlińska et al., 2013, 2015). The same video showing a case of cyberbullying was used as the basis for the “empathy activating task”. The main modification, intended to activate the process of cognitive empathy, was asking the experimental participants to select, from a list of possible emotions, which feelings the victim conveyed in the recording. Specifically, before viewing the film, the students were told to concentrate on how the victim might feel and try to identify with the situation depicted, focusing on those aspects that reflected her emotions. Afterwards, the participants checked off from a multiple-choice list those emotions that appeared in the video. The list comprised both adequate emotion labels (demonstrated or stated by the actress in the movie) and inadequate (not present in the film). Selecting the wrong set of answers was followed by an instruction, “Please try again to select the correct answers”. Three trials were available. The number of trials was a controlled variable operationalizing repetitiveness (perceived as an important condition of effectiveness of cognitive empathy induction). The set of correct and incorrect answers is based on the results of a pilot study on 80 junior high school students – the five most commonly cited characteristics of feelings were used for the correct answers set, and two randomly selected were used for the incorrect set. These are presented in **Table 3**.

**Table 3:** Correct and incorrect answers in the cognitive empathy activation condition.

Correct	Incorrect
Fear	Satisfaction
Anger	nothing special
Injustice	
Shame	
Harm	

In the control condition, the task was to answer the question, “Where is the action movie set?” focusing on the elements of the background and selecting scenes that appeared in the video from a longer list presented in **Table 4**.

**Table 4:** Correct and incorrect answers in the control condition.

Correct	Incorrect
In the girl’s room	On the street
On the computer screen	In church
On the mobile phone screen	
In the gym	
In the school locker room	

For control purposes, in both conditions the number of trials was recorded and used in the analysis.

### Plan of Analysis

As in the first study logistic regression analysis was performed with SPSS 25. The analysis was conducted to evaluate whether activating cognitive empathy would increase the likelihood of intervening cyberbystander behavior. The impact of gender, cyberperpetration and cybervictimization on cyberbystander behavior was analyzed. Additionally, the number of attempts in giving correct answers and previous viewing of the film was controlled.

### Results

We conducted logistic regression analysis to determine the impact of cognitive empathy activation on bystander helping behavior. There were no missing values and all analyses were conducted on the full sample. The analysis showed that the model fit the data well and was significant. Overall statistics were significant; further, pseudo R squares, indicating the amount of explained variance, were substantial (**Table 5**).

**Table 5:** The results of the logistic regression analysis for activation of cognitive empathy, cyberperpetration, cybervictimization, gender, previous viewing the film, and number of trails on intervening cyberbystander behavior.

Predictor	*B*	*SE B*	Wald’s χ^2^	OR [CI95%]
Gender (0 – boys)	0.06	0.31	0.04	1.06 [0.57–1.96]
Control film – yes (0 – didn’t see)	2.02	0.49	16.97^∗∗∗^	7.54 [2.88–19.73]
Control film – don’t remember (0 – didn’t see)	1.00	0.35	8.27^∗∗^	2.72 [1.37–5.39]
No of trials	0.54	0.13	16.89^∗∗∗^	1.71 [1.37–5.39]
Cyberperpetration	-0.03	0.28	0.01	0.97 [0.56–1.67]
Cybervictimization	-0.33	0.35	0.89	0.76 [0.36–1.43]
Condition (0 – no empathy)	1.86	0.35	28.46^∗∗∗^	6.41 [3.24–12.67]

**Overall model**			**χ^2^**	

Likelihood ratio test			50,27^∗∗∗^	
Score test			54,31^∗∗∗^	
HandL			8,85


*^∗^*p* < 0.05, ^∗∗^*p* < 0.01,^∗∗∗^*p* < 0.001.*

*Step 1: Cox and Snell *R*^*2*^ = 0.19; Nagelkerke *R*^*2*^ = 0.25*.

Similar to Study 1, gender and prior cyberperpetration and cybervictimization were found to be insignificant. Two of the controls were found to be significant. First, those participants who had previously seen the film chose to intervene seven and one-half times more often than those who had not seen the movie. Similarly, the group of participants who did not remember if they had seen the movie still chose the intervening cyberbystander behavior almost three times more often those who viewed it for the first time. Second, the number of trials also proved to be significant. The effect shows that, with every single attempt, the probability of choosing helpful behavior increased almost twofold.

Our main result shows that cognitive empathy activation has a significant and substantial effect on increasing the tendency to report the abuse. Participants in the experimental condition, in which cognitive empathy was activated, were six and one half times more likely to choose a helping reaction than participants in the control condition. It is worth mentioning that this effect was independent of all other controls.

### Discussion

Active taking of the perspective of a cyberbullying victim proved to significantly increase the probability of reporting abuse by bystanders to cyberbullying. The results of this study confirm previous findings indicating that cognitive empathy is a significant factor related to both offline (Hoffman, 2000; Nickerson et al., 2008; Caravita et al., 2009) and online helping behavior (Macháčková et al., 2013; Schultze-Krumbholz et al., 2016). Other effects obtained in the first study have been replicated: gender, cybervictimization, cyberperpetration did not increase the intervening cyberbystander behavior.

Independent, significant effects of previous viewing of the film and the number of attempts giving correct answers significantly increased the probability of reporting cyberbullying by bystanders. These results are in line with data showing that longer and repetitive forms of intervention intensify reflective information processing and, consequently, increase its effectiveness (Hoffman, 2000; Schultze-Krumbholz et al., 2016). They also confirm the effectiveness of strategies focusing the cyberbystander on the victim’s perspective (Macháčková et al., 2016). Additionally, the effect of number of trials is most probably an outcome of deeper processing of the manipulation material, thereby enforcing the impact of the manipulation. Yet, the independent nature of the effects of empathy activation and the number of trials justify an interpretation in terms of additive influences of cognitive empathy and reflectiveness, with the latter being the result of longer concentration on content related to emotional consequences of cybervictimization. A good explanation for this phenomenon is found in social learning theory (Bandura, 1973); this model is consistent with the need for a repetitive and longer form of empathy training to effectively reduce cyberbullying behavior.

These considerations suggest a deeper understanding of the other person’s situation can encourage prosocial online behavior such as reporting cyberbullying acts.

## General Discussion

Cyberbullying, with its own specific features, requires different modes of effective intervention than those that apply to face-to-face bullying (Ttofi and Farrington, 2011; Ang, 2015; Nocentini et al., 2015). It is of paramount importance to identify factors that not only reduce cyberbullying acts but, first and foremost, lead to intensify proactive behavior (i.e., reporting the negative behaviors). The present research focused on cyberbystander behavior which was the effect of the decision: what to do with online content that is harmful to a peer? Reporting such abuse in a situation where one’s psychological well-being and fundamental norms are being violated may be viewed as civil courage (Livingstone et al., 2011). Relevant research clearly states what kind of dispositional correlates are connected to prosocial online behavior: well developed social skills, low levels of moral disengagement, high social self-efficacy and high levels of both affective and cognitive empathy (Gini et al., 2007, 2008; Nickerson et al., 2008; Menesini et al., 2012). Cognitive empathy is one of these individual dispositions that could be effectively trained. Results of research on empathy development emphasize the significance of both parental and school impact. Manifestation of sorrow or joy in reaction to child’s behaviors and, first and foremost, directing a child’s attention to the impact of his/her behaviors upon others reinforces emphatic response mechanisms (Hoffman, 2000).

In the present study, focused on raising the chances for bystander intervention in cases of cyberbullying, only cognitive empathy activation proved to be effective. The limitations of affective empathy induction on prosocial bystander behavior also have been revealed. The obtained results confirm that cognitive empathy is one of those determinants which can be effectively activated, even in the form of a brief intervention, stimulating cyberbystander intervening reactions to cyberbullying. Also, higher effectiveness of repetitive induction has been confirmed. Our results are coherent and consistent with others concerning: (a) the role of perspective taking (Batson, 1991, 2011; Eisenberg, 2000; Hoffman, 2000); (b) the relationship of cognitive empathy to cyberbullying (Steffgen et al., 2011; Pfetsch and Ittel, 2014) and, especially, (c) the association of cognitive empathy and cyberbystander responses (Barlińska et al., 2013, 2015; Freis and Gurung, 2013; Macháčková et al., 2013). They also support current knowledge on the importance of situational factors in determining whether a person intervenes in a cyberbullying incident (DeSmet et al., 2016; Macháčková et al., 2016; Pfetsch, 2016). In particular, they highlight the role of situational cognitive empathy priming, as may increase availability (and thus awareness) of the other person’s perspective (De Vignemont, 2006).

Several conclusions follow the results of this study. First, the findings show the complexity of the relationship between activated empathy and prosocial and antisocial behavior. Contrary to the results of previous research on the effectiveness of both affective and cognitive empathy in limiting pro-bullying cyberbystander behavior (Barlińska et al., 2013), only cognitive empathy induction was found to significantly increase helping cyberbystander behavior. These results are consistent with some scarce data (e.g., Krueger et al., 2001) suggesting that altruism and antisocial behavior are uncorrelated tendencies stemming from different sources. That is, activating prosocial (reporting the abuse) and reinforcing (sending on) cyberbystander behavior are not simply mirror effects. Rather, based on our findings, the circumstances leading to their activation may be distinct. Further, considering the obtained results in context of the phenomenon of empathy appears particularly worthy, as they corroborate its complex and multidimensional nature. They also confirm Bloom’s hypothesis suggesting that empathy and concern are psychologically distinct, with empathy (in our terms, the affective dimension) playing a more limited role in people’s moral choices than commonly thought.

Why was affective empathy induction found to be an ineffective prosocial behavior strategy? According to the perception–action model of empathy (Preston and De Waal, 2002), related to affective empathy, merely observing what the other person feels automatically triggers the neural pathways which evoke the same affective states as those evoked in that other. On an unconscious level, it is possible to detect another’s state and react in a syntonic way, even if we are unaware of our own feelings. If we recognize the other’s pain or joy, we can also automatically react to it by feeling the same. Such shared emotions can lead to appraisal of the other’s situation and deciding how to respond. Does such interpersonal transmission lead to positive consequences for the other person? In most cases syntonic reactions are considered positive from the standpoint of that other. But in the case of negative affect, contagion can lead to negative consequences for both parties. Feedback from the observer, moreover, can increase the subject’s anxiety. The observer who feels discomfort may try to keep his/her distance or may respond in a negative, even aggressive way. Further, affective empathy activates the automatic channel of behavior regulation. The option of sharing experiences with friends is more consistent with the automatic mode than is reporting abuse. This is particularly seen in adolescents (DeSmet et al., 2016). It is a behavior pattern that is repeated numerous times, an element of a universal adolescent online functioning script. Thus the processing mode induced by affective empathy facilitating automatic script-like behaviors may, paradoxically, create a preference for sharing cyberbullying acts more than reporting them as abuse.

Why was cognitive empathy found to compensate for affective empathy deficits in inducing prosocial behaviors? According to the social cognitive neuroscience model of human empathy (Decety, 2007), the empathy arises as a result of dynamic interaction of the following four functional elements: (a) affect-sharing between the self and others; (b) self-awareness and self-other differentiation; (c) the subject’s mental flexibility to adopt the perspective of the other and, lastly, (d) regulatory processes, including emotion regulation. Cognitive empathy—built upon an appreciation of another’s situation and needs—is connected with a person’s favorable affects and behavior. Its two fundamental features are: (a) the capacity for conscious recognition, and (b) reflective appraisal of the other’s state or situation. It requires involvement of complex cognitive and evaluative processes like perspective taking (Batson et al., 1997). Behaviors are strictly related to one’s concentration on the other person; the accompanying emotions are of post-cognitive nature. This is conducive to effective emotion regulation and increased behavior control. As a result, it makes cognitive empathy-motivated involvement more suitable for the online environment.

These presumptions about the specific mechanisms and strategy determinants of effective empathy activation on cyberbystander behavior are in line with some scarce data on the effectiveness of interventions geared specifically toward the online context. DeSmet et al. (2018), for example, concluded that empathy training was needed to achieve a change in negative cyberbystander behavior. The effectiveness of our proposed method of activating empathy should be further tested in a comprehensive school program, not a single component study (as currently presented). An approach exploring the effectiveness of empathy activation in various relationships that proved to affect bullying – peer and student–teacher, should be tested (Longobardi et al., 2018). Ttofi and Farrington (2011) clearly stated the need for theoretically grounded and rigorously implemented and evaluated programs to prevent cyberbullying. Until now, most studies on evidence- and school-based anti-cyberbullying programs focused mostly on cybervictims and cyberbullies. Despite the growing attention on cyberbystanders, there still are knowledge gaps regarding which interventions will encourage prosocial online responses (through effective situational activation of factors leading to same). The current research fills this gap, adding conclusions for prevention of antisocial online behavior. In sum, results of the current studies suggest that actively taking the perspective of the cybervictim (cognitive empathy) can lead to more interventions and fewer passive reactions in cyberbystanders. To achieve such results in school practice, educators need to implement focused cognitive empathy-activating tasks. These can enhance students’ empathy and encourage prosocial bystander responses, especially for those likely to be involved in reinforcing cyberbullying.

The current investigation has its strengths and limitations. The main strength is the general design using an experimental approach with video clips, due to the ecological appropriateness and attractiveness to the studied group: adolescents. On the other hand, the main limitations are, to a degree, a consequence of the methodological approach: the obtained results were gathered from a purposefully recruited sample. Future research on effectiveness of empathy induction on cyberbystander behavior should collect data from a randomly selected sample. Also, the impact of order effects should be considered in further research. Additionally, only one of several possible prosocial reactions, reporting the abuse, has been tested. A broader set of potential responses (e.g., defending, comforting) could yield valuable insight. In line with this concern, a conclusion of both ineffectiveness of affective and effectiveness of cognitive empathy activation is constrained to this specific form of prosocial online behavior. Additionally, the severity of the cyberbullying act was not differentiated. The bullying behavior witnessed by the participants is a relatively mild form that may restrict generalizing our findings to more or less severe forms of cyberbullying.

Notwithstanding these limitations, our results suggest that cognitive empathy focuses a person’s attention on the external situation of another person. This, in turn, activates prosocial behavior mechanisms aimed at improving the predicament of the other without expecting any external reinforcements (Berkowitz and Macaulay, 1970). Thereby, it justifies the finding that cognitive empathy leads to more selective and insightful perceiving of social situations in cases where even such slight symptom of cyber-aggression can prompt a helping reaction.

The educational recommendations provided herein require further exploration in a more complex study on the effectiveness of a holistic, evidence-based anti-cyberbullying program. Such a context should include activities aimed at inducing cognitive empathy, as may give rise to alternative, prosocial activities in cyberbystanders. Future research and interventions should take into account the complex nature of the mechanisms of empathy induction in a more holistic school based approach. These may require different actions to effectively trigger prosocial, and diminish antisocial, cyberbystander behavior.

## Author Contributions

JB contributed to conducting the research, the theoretical part on cyberbullying and cyberbystanders, and to the description of the procedure, methods, and discussion. AS contributed to the theoretical part on empathy and to the discussion. MW contributed to the analysis.

## Conflict of Interest Statement

The authors declare that the research was conducted in the absence of any commercial or financial relationships that could be construed as a potential conflict of interest.

## Footnotes

1 http://www.saferinternet.pl/about-us.html

2 The effectiveness of the empathy manipulation procedure was previously established in a pilot study using a Polish adaptation of the Positive and Negative Affect Schedule (PANAS; Watson et al., 1988; Barlińska et al., 2013).

## References

[B1] Ang R. P. (2015). Adolescent cyberbullying: a review of characteristics, prevention and intervention strategies. *Aggress. Violent Behav.* 25 35–42. 10.1016/j.avb.2015.07.011

[B2] Bandura A. (1973). *Aggression: A Social Learning Analysis.* Englewood Cliffs, NJ: Prentice-Hall.

[B3] Barlett C. Coyne S. M. (2014). A meta-analysis of sex differences in cyber-bullying behavior: the moderating role of age. *Aggress. Behav.* 40 474–488. 10.1002/ab.21555 25098968

[B4] Barlinìska J. Wojtasik Ł. (2008). “Peer violence and electronic media – Research and social campaign,” in *Teenagers’ Actions and Interactions Online in Central and Eastern Europe. Potentials and Empowerment, Risks and Victimization*, eds Barbovschi M. Diaconescu M. (Cluj-Napoca: Cluj University Press), 281–299.

[B5] Barlińska J. Szuster A. Winiewski M. (2013). Cyberbullying among adolescent bystanders: role of the communication medium, form of violence, and empathy. *J. Community Appl. Soc. Psychol.* 23 37–51. 10.1002/casp.2137

[B6] Barlińska J. Szuster A. Winiewski M. (2015). The role of short-and long-term cognitive empathy activation in preventing cyberbystander reinforcing cyberbullying behavior. *Cyberpsychol. Behav. Soc. Netw.* 18 241–244. 10.1089/cyber.2014.0412 25802977

[B7] Baron-Cohen S. (2011). *The Science of Evil.* London: Basic Book.

[B8] Bastiaensens S. Vandebosch H. Poels K. Van Cleemput K. De Smet A. De Bourdeaudhuij I. (2014). Cyberbullying on social network sites. An experimental study into bystanders’ behavioural intentions to help the victim or reinforce the bully. *Comput. Hum. Behav.* 31 259–271. 10.1016/j.chb.2013.10.036

[B9] Batson C. D. (1991). *The Altruism Question: Toward a Social-Psychological Answer.* Hillsdale, NJ: Erlbaum Associates.

[B10] Batson C. D. (2011). *Altruism in Human.* Oxford: Oxford University Press.

[B11] Batson C. D. Ahmad N. (2001). Empathy-induced altruism in a Prisoner’s Dilemma II: what if the target of empathy has defected? *Eur. J. Soc. Psychol.* 31 25–36. 10.1002/ejsp.26

[B12] Batson C. D. Ahmad N. (2009). Using empathy to improve intergroup attitudes and relations. *Soc. Issues Policy Rev.* 3 141–177. 10.1111/j.1751-2409.2009.01013 25760634

[B13] Batson C. D. Early S. Salvarani G. (1997). Perspective taking: imagining how another feels versus imagining how you would feel. *Pers. Soc. Psychol. Bull.* 23 751–758. 10.1177/0146167297237008 16140345

[B14] Batson D. C. Fultz J. Schoenrade P. A. (1987). Distress and empathy: two qualitatively distinct vicarious emotions with different motivational consequences. *J. Pers.* 55 19–39. 10.1111/j.1467-6494.1987.tb00426 3572705

[B15] Batson C. D. Shaw L. L. (1991). Evidence for altruism: toward a pluralism of prosocial motives. *Psychol. Inq.* 2 107–122. 10.1207/s15327965pli0202_1

[B16] Beran T. Li Q. (2007). The relationship between cyberbullying and school bullying. *J. Student Wellbeing* 1 16–33. 10.21913/JSW.v1i2.172

[B17] Berkowitz L. Macaulay J. R. (1970). *Altruism and Helping Behavior.* New York, NY: Academic Press.

[B18] Bloom P. (2016). *Against Empathy.* New York, NY: HarperCollins Publisher.

[B19] Caravita S. DiBlasio P. Salmivalli C. (2009). Unique and interactive effects of empathy and social status on involvement in bullying. *Soc. Dev.* 18 140–163. 10.1111/j.1467-9507.2008.00465.x

[B20] Chalmers J. B. Townsend R. (1990). The effect of training in social perspective taking on socially maladjusted girls. *Child Dev.* 61 178–190. 10.2307/1131057 2307038

[B21] Chandler M. J. (1973). Egocentric and antisocial behaviour. The assessment and training of social and perspective-taking skills. *Dev. Psychol.* 9 326–332. 10.1037/h0034974

[B22] Cook C. R. Williams K. R. Guerra N. G. Kim T. E. (2010). “Variability in the prevalence of bullying and victimization: a cross-national and methodological analysis,” in *Handbook of Bullying in Schools: An International Perspective*, eds Jimerson S. Swearer S. M. Espelage D. L. (London: Routledge), 347–362.

[B23] Davis M. H. (1996). *Empathy: A Social-Psychological Approach.* Boulder, CO: Westview Press.

[B24] Davis M. H. Conklin L. Smith A. Luce C. (1996). Effect of perspective taking on cognition representation of persons: a merging of self and other. *J. Pers. Soc. Psychol.* 70 713–726. 10.1037/0022-3514.70.4.713 8636894

[B25] De Vignemont F. (2006). “When do we empathize?,” in *Empathy and Fairness*, ed. C. Frith (New York, NY: John Wiley & Sons), 180–195.

[B26] De Vignemont F. Singer T. (2006). The empathic brain: how, when and why? *Trends Cogn. Sci.* 10 435–441. 10.1016/j.tics.2006.08.008 16949331

[B27] Decety J. (2007). “A social cognitive neuroscience model of human empathy,” in *Social Neuroscience*, eds Harmon-Jones E. Winkielman P. (New York, NY: The Guilford Press), 246–269.

[B28] Decety J. Jackson P. L. (2004). The Functional architecture of human empathy. *Behav. Cogn. Neurosci. Rev.* 3 71–100. 10.1177/1534582304267187 15537986

[B29] Dehue F. Bolman C. Völlink T. (2008). Cyberbullying: youngsters’ experiences and parental perception. *Cyberpsychol. Behav.* 11 217–223. 10.1089/cpb.2007.0008 18422417

[B30] Dehue F. Bolman C. Völlink T. Pouwelse M. (2012). Cyberbullying and traditional bullying in relation with adolescents’ perception of parenting. *J. Cyberther. Rehabil.* 5 25–34.

[B31] DeSmet A. Bastiaensens S. Van Cleemput K. Poels K. (2016). Deciding whether to look after them, to like it, or leave it: a multidimensional analysis of predictors of positive and negative bystander behavior in cyberbullying among adolescents. *Comp. Hum. Behav.* 57 398–415. 10.1016/j.chb.2015.12.051

[B32] DeSmet A. Bastiaensens S. Van Cleemput K. Poels K. Vandebosch H. De Bourdeaudhuij I. (2012). Mobilizing bystanders of cyberbullying: an exploratory study into behavioural determinants of defending the victim. *Stud. Health Technol. Inform.* 181 58–63. 22954829

[B33] DeSmet A. Bastiaensens S. Van Cleemput K. Poels K. Vandebosch H. Deboutte G. (2018). The efficacy of the Friendly Attac serious digital game to promote prosocial bystander behavior in cyberbullying among young adolescents: a cluster-randomized controlled trial. *Comp. Hum. Behav.* 78 336–347. 10.1016/j.chb.2017.10.011

[B34] DeSmet A. Veldeman C. Poels K. Bastiaensens S. Van Cleemput K. Vandebosch H. (2014). Determinants of self-reported bystander behavior in cyberbullying incidents amongst adolescents. *Cyberpsychol. Behav. Soc. Netw.* 17 207–215. 10.1089/cyber.2013.0027 24359305

[B35] Dillon K. P. Bushman B. J. (2015). Unresponsive or un-noticed?: cyberbystander intervention in an experimental cyberbullying context. *Comp. Hum. Behav.* 45 144–150. 10.1016/j.chb.2014.12.009

[B36] Eisenberg N. (2000). Emotion, regulation and moral development. *Ann. Rev. Psychol.* 51 665–697. 10.1146/annurev.psych.51.1.665 10751984

[B37] Eisenberg N. Miller P. A. (1987). Empathy and prosocial behaviour. *Psych. Bull.* 101 91–119. 10.1037/0033-2909.101.1.9 3562705

[B38] Eisenberg N. Morris A. S. (2001). The origins and social significance of empathy-related responding. *Soc. Justice* 14 95–120. 10.1023/A:1012579805721

[B39] Eisenberg N. Strayer J. (1987). “Critical issues in the study of empathy,” in *Empathy and Its Development*, eds Eisenberg N. Strayer J. (Cambridge: Cambridge University Press), 3–13.

[B40] Erdur-Baker O. (2010). Cyberbullying and its correlation to traditional bullying, gender, and frequent and risky usage of internet-mediated communication tools. *New Media Soc.* 21 109–125. 10.1177/1461444809341260

[B41] Fawzi N. Goodwin B. (2011). Witnesses of the offense: what influences the behavior of bystanders of cyberbullying? *Paper Presented at the Annual Meeting of the International Communication Association, TBA*, Boston, MA.

[B42] Fletcher A. Fitzgerald-Yau N. Jones R. Allen E. Viner R. M. Bonell C. (2014). Brief report: cyberbullying perpetration and its associations with socio-demographics, aggressive behaviour at school, and mental health outcomes. *J. Adolesc.* 37 1393–1398. 10.1016/j.adolescence.2014.10.005 25448835

[B43] Freis S. D. Gurung R. A. (2013). A Facebook analysis of helping behavior in online bullying. *Psychol. Pop. Media Cult.* 2 11–19. 10.1037/a0030239

[B44] Gini G. Albiero P. Benelli B. Altoè G. (2007). Does empathy predict adolescents’ bullying and defending behavior? *Aggress. Behav.* 33 467–476. 10.1002/ab.20204 17683107

[B45] Gini G. Albiero P. Benelli B. Altoe G. (2008). Determinants of adolescents’ active defending and passive bystanding behavior in bullying. *J. Adolesc.* 31 93–105. 10.1016/j.adolescence.2007.05.002 17574660

[B46] Hinduja S. Patchin J. W. (2013). Social influences on cyberbullying behaviors among middle and high school students. *J. Youth Adolesc.* 42 711–722. 10.1007/s10964-012-9902-4 23296318

[B47] Hoffman M. L. (2000). *Empathy and Moral Development. Implication for Caring and Justice.* Cambridge: Cambridge University Press 10.1017/CBO9780511805851

[B48] Hoffman L. M. (2008). “Empathy and prosocial behavior,” in *Handbook of Emotions*, eds Lewis M. Haviland-Jones J. M. Barrett L. Feldman (New York, NY: The Guilford Press), 440–454.

[B49] Hogan R. (1969). Development of an empathy scale. *J. Consult. Clin. Psychol.* 33 307–316. 10.1037/h0027580 4389335

[B50] Jordan M. Amir D. Bloom P. (2016). Are empathy and concern psychologically distinct? *Emotion* 16 1107–1116. 27668802 10.1037/emo0000228

[B51] Juvonen J. Gross E. F. (2008). Extending the school grounds? Bullying experiences in cyberspace. *J. Sch. Health* 78 496–505. 10.1111/j.1746-1561.2008.00335 18786042

[B52] Kiesler S. Siegel J. McGuire T. W. (1984). Social psychological aspects of computer mediated communications. *Am. Psychol.* 39 1123–1134. 10.1037/0003-066X.39.10.1123

[B53] Kowalski R. M. Giumetti G. W. Schroeder A. N. Lattaner M. R. (2014). Bullying in the digital age: a critical review and meta-analysis of cyberbullying research among youth. *Psychol. Bull.* 140 1073–1137. 10.1037/a0035618 24512111

[B54] Krueger R. Hicks B. M. McGue M. (2001). Altruism and antisocial behavior: independent tendencies, unique personality correlates, distinct etiologies. *Psychol. Sci.* 12 397–402. 10.1111/1467-9280.00373 11554673

[B55] Lamm C. Batson C. D. Decety J. (2007). The neural substrate of human empathy: effects of perspective-taking and cognitive appraisal. *J. Cogn. Neurosci.* 19 42–58. 10.1162/jocn.2007.19.1.42 17214562

[B56] Latané B. Darley J. M. (1970). *The Unresponsive Bystander: Why Doesn’t He Help.* New York, NY: Appleton-Century-Crofts.

[B57] Lerner M. J. (1977). The justice motive in social behavior: some hypotheses to its origins and forms. *J. Pers.* 45 1–52. 10.1111/j.1467-6494.1977.tb00591.x

[B58] Li Q. (2006). Cyberbullying in schools a research of gender differences. *Sch. Psychol. Int.* 27 157–170. 10.1177/0143034306064547

[B59] Livingstone S. Haddon L. Görzig A. Ólafsson K. (2011). *Risks and Safety on the Internet: The Perspective of European Children. Full Findings.* London: EU Kids Online.

[B60] Longobardi C. Iotti N. O. Jungert T. Settanni M. (2018). Student-teacher relationships and bullying: the role of student status. *J. Adolesc.* 63 1–10. 10.1016/j.adolescence.2017.12.001 29222968

[B61] Macaula P. Boulton M. J. (2017). “Adolescent bystander responses to offline and online bullying: the role of bullying severity and empathy,” in *Proceedings of the 22nd Annual CyberPsychology, CyberTherapy & Social Networking Conference*, University of Wolverhampton, Wolverhampton.

[B62] Macháčková H. Dedkova L. Mezulanikova K. (2015). The bystander effect in cyberbullying incidents. *J. Adolesc.* 43 96–99. 10.1016/j.adolescence.2015.05.010 26070168

[B63] Macháčková H. Dedkova L. Sevcikova A. Cerna A. (2013). Bystanders’ support of cyberbullied schoolmates. *J. Commun. Appl. Soc. Psychol.* 23 25–36. 10.1002/casp.2135

[B64] Macháčková H. Dedkova L. Sevcikova A. Cerna A. (2016). Empathic responses by cyberbystanders: the importance of proximity. *J. Youth Stud.* 19 793–804. 10.1080/13676261.2015.1112882

[B65] Matthews K. A. Batson C. D. Horn J. Rosenman R. H. (1981). Principles in his nature which interest him in the fortune of other: the heritability of empathic concern for others. *J. Pers.* 49 237–247. 10.1111/j.1467-6494.1981.tb00933

[B66] Menesini E. Codecasa E. Benelli B. (2003). Enhancing children’s responsibility to take action against bullying: evaluation of a befriending intervention in Italian middle schools. *Aggress. Behav.* 29 10–14. 10.1002/ab.80012

[B67] Menesini E. Eslea M. Smith P. K. Genta M. L. Giannetti E. Fonzi A. (1997). Cross-national comparison of children’s attitudes towards bully/victim problems in schools. *Aggress. Behav.* 23 245–257. 10.1002/(SICI)1098-2337(1997)23:4<245::AID-AB3>3.0.CO;2-J

[B68] Menesini E. Nocentini A. Palladino B. Frisén A. Berne S. Ortega-Ruiz R. (2012). Cyberbullying definition among adolescents: a comparison across six European countries. *Cyberpsychol. Behav. Soc. Netw.* 15 455–463. 10.1089/cyber.2012.0040 22817693 PMC3443335

[B69] Modecki K. L. Minchin J. Harbaugh A. G. Guerra N. G. Runions K. C. (2014). Bullying prevalence across contexts: a meta-analysis measuring cyber and traditional bullying. *J. Adolesc. Health* 55 602–611. 10.1016/j.jadohealth.2014.06.007 25168105

[B70] Nickerson A. B. Mele D. Princiotta D. (2008). Attachment and empathy as predictors of roles as defenders or outsiders in bullying interactions. *J. Sch. Psychol.* 46 687–703. 10.1016/j.jsp.2008.06.002 19083379

[B71] Nocentini A. Zambuto V. Menesini E. (2015). Anti-bullying programs and information and communication technologies (ICTs): a systematic review. *Aggress. Violent Behav.* 23 52–60. 10.1016/j.avb.2015.05.012

[B72] O’Neill B. McLaughlin S. (2010). *Recommendations on Safety Initiatives.* London: EU Kids Online.

[B73] Obermaier M. Fawzi N. Koch T. (2014). Bystanding or standing by? How the number of bystanders affects the intention to intervene in cyberbullying. *New Media Soc.* 18 1491–1507. 10.1177/1461444814563519

[B74] Oh I. Hazler R. J. (2009). Contributions of personal and situational factors to bystanders’ reactions to school bullying. *Sch. Psychol. Int.* 30 291–310. 10.1177/0143034309106499

[B75] Olweus D. (2001). “Peer harassment. A critical analysis and some important issues,” in *Peer Harassment in School. The Plight of the Vulnerable and Victimized*, eds Juvonen J. Graham S. (New York, NY: Guilford Press), 3–20.

[B76] Olweus D. (2012). Cyberbullying: an overrated phenomenon? *Eur. J. Dev. Psychol.* 9 520–538. 10.1080/17405629.2012.682358

[B77] Ortega-Ruiz R. Del Rey R. Casas J. A. (2012). Knowing, building and living together on internet and social networks: the ConRed cyberbullying prevention program. *Int. J. Confl. Violence* 6 303–312. 10.4119/UNIBI/ijcv.250

[B78] Palladino B. E. Menesini E. Nocentini A. Luik P. Naruskov K. Ucanok Z. (2017). Perceived severity of cyberbullying: differences and similarities across four countries. *Front. Psychol.* 8:1524. 10.3389/fpsyg.2017.01524 28979217 PMC5611493

[B79] Palladino B. E. Nocentini A. Menesini E. (2012). Online and offline peer led models against bullying and cyberbullying. *Psicthema* 24 634–639. 23079363

[B80] Peng C.-Y. J. Lee K. L. Ingersoll G. M. (2002). An introduction to logistic regression analysis and reporting. *J. Educ. Res.* 96 3–14. 10.1080/00220670209598786

[B81] Pfetsch J. (2016). “Chapter 9: who is who in cyberbullying? Conceptual and empirical perspectives on bystanders in cyberbullying,” in *A Social-Ecological Approach to Cyberbullying*, ed. M. F. Wright (Hauppauge, NY: Nova Publishing), 121–150.

[B82] Pfetsch J. (2017). Empathic skills and cyberbullying: relationship of different measures of empathy to cyberbullying in comparison to offline bullying among young adults. *J. Genet. Psychol.* 178 1 58–72. 10.1080/00221325.2016.1256155 28121287

[B83] Pfetsch J. Ittel A. (2014). “Adolescent bystanders of cyberbullying – traditional bullying, empathy, and parental mediation as correlates of online behavior,” in *Poster Presentation, 15th Biennial Meeting of the Society for Research on Adolescence (SRA)*, Austin, TX.

[B84] Piliavin J. A. Dovidio J. F. Gaertner S. L. Clark R. D. (1982). “Responsive bystanders: the process of intervention,” in *Cooperation and Helping Behavior*, eds Derlega J. V. Grzelak J. (New York, NY: Academic Press), 279–304. 10.1016/B978-0-12-210820-4.50017-4

[B85] Pornari C. D. Wood J. (2010). Peer and cyber aggression in secondary school students: the role of moral disengagement, hostile attribution bias, and outcome expectancies. *Aggress. Behav.* 36 81–94. 10.1002/ab.20336 20035548

[B86] Preston S. D. De Waal F. (2002). Empathy: its ultimate and proximate bases. *Behav. Brain Sci.* 25 1–72. 12625087 10.1017/s0140525x02000018

[B87] Pyżalski J. (2013). Beyond peer cyberbullying – involvement of polish adolescents in different kinds of electronic aggression. *Stud. Eduk.* 28 147–168.

[B88] Quirk R. Campbell M. (2014). On standby? A comparison of online and offline witnesses to bullying and their bystander behaviour. *Educ. Psych. Int. J. Exp. Educ. Psychol.* 35 430–448. 10.1080/01443410.2014.893556

[B89] Raskauskas J. Stoltz A. D. (2007). Involvement in traditional and electronic bullying among adolescents. *Dev. Psychol.* 43 564–575. 10.1037/0012-1649.43.3.564 17484571

[B90] Rigby K. Slee P. T. (1991). Bullying among Australian schoolchildren: reported behavior and attitudes toward victims. *J. Soc. Psychol.* 131 615–627. 10.1080/00224545 1798296

[B91] Rushton J. P. Fulker D. W. Neale M. C. Nias D. K. B. Eysenck H. J. (1986). Altruism and aggression: the heritability of individual differences. *J. Pers. Soc. Psychol.* 50 1192–1198. 10.1037/0022-3514.50.6.1192 3723334

[B92] Salmivalli C. (2010). Bullying and the peer group: a review. *Aggress. Violent Behav.* 15 112–120. 10.1016/j.avb.2009.08.007

[B93] Salmivalli C. Lagerspetz K. Bjorkqvist K. Osterman K. Kaukiainen A. (1996). Bullying as a group process: participant roles and their relations to social status within the group. *Aggress. Behav.* 22 1–15. 10.1002/(SICI)1098-2337(1996)22:1<1::AID-AB1>3.0.CO;2-T

[B94] Salmivalli C. Voeten M. Poskiparta E. (2011). Bystanders matter: associations between reinforcing, defending, and the frequency of bullying behavior in classrooms. *J. Clin. Child Adolesc. Psychol.* 40 668–676. 10.1080/15374416.2011.597090 21916686

[B95] Schultze-Krumbholz A. Schultze M. Zagorscak P. Wölfer R. Scheithauer H. (2016). Feeling cybervictims’ pain—The effect of empathy training on cyberbullying. *Aggress. Behav.* 42 147–156. 10.1002/ab.21613 26349848

[B96] Shamy-Tsoory S. G. Aron-Peretz J. Perry D. (2009). Two systems for empathy: a double dissociation between emotional and cognitive empathy in inferior frontal gyrus versus ventromedial prefrontal lesions. *Brain* 132 617–627. 10.1093/brain/awn279 18971202

[B97] Shultz E. Heilman R. Hart K. J. (2014). Cyber-bullying: an exploration of bystander behavior and motivation. *Cyberpsychology* 8:3 10.5817/CP2014-4

[B98] Smith A. (1759/1976). *The Theory of Moral Sentiments.* Oxford: Clarendon Press.

[B99] Smith P. K. (2016). Bullying: definition, types, causes, consequences and intervention. *Soc. Pers. Psychol. Compass* 10/9 519–532. 10.1111/spc3.12266

[B100] Smith P. K. Kwak K. Toda Y. (2016). *School Bullying in Different Cultures: Eastern and Western Perspectives.* Cambridge, MA: Cambridge University Press 10.1017/CBO9781139410878

[B101] Smith P. K. Mahdavi J. Carvalho M. Fisher S. Russell S. Tippett N. (2008). Cyberbullying, its forms and impact in secondary school pupils. *J. Child Psychol. Psychiatry* 49 376–385. 10.1111/j.1469-7610.2007.01846.x 18363945

[B102] Song J. Oh I. (2018). Factors influencing bystanders’ behavioral reactions in cyberbullying situations. *Comp. Hum. Behav.* 78 273–282. 10.1016/j.chb.2017.10.008

[B103] Steffgen G. König A. Pfetsch J. Melzer A. (2011). Are cyberbullies less empathic? Adolescents’ cyberbullying behavior and empathic responsiveness. *Cyberpsychol. Behav. Soc. Netw.* 14 643–648. 10.1089/cyber.2010.0445 21554126

[B104] Sticca F. Ruggieri S. Alsaker F. Perren S. (2013). Longitudinal risk factors for cyberbullying in adolescence. *J. Comm. Appl. Soc. Psychol.* 23 52–67. 10.1002/casp.2136

[B105] Stone V. Baron-Cohen S. Knight K. (1998). Frontal lobe contributions to theory of mind. *J. Cogn. Neurosci.* 10 640–656. 10.1162/089892998562942 9802997

[B106] Szuster A. (2016). Crucial dimensions of human altruism. Affective vs. conceptual factors leading to helping or reinforcing others. *Front. Psychol.* 7:519. 10.3389/fpsyg.2016.00519 27148127 PMC4829577

[B107] Szuster A. Barlińska J. Kozubal M. (2016). “In search of a simple method: is a human face an effective, automatic filter inhibiting cyberbullying?,” in *A Social-Ecological Approach to Cyberbullying*, ed. M. F. Wright (Hauppauge, NY: Nova Publishing), 379–402.

[B108] Ttofi M. M. Farrington D. P. (2011). Effectiveness of school-based programs to reduce bullying: a systematic and meta-analytic review. *J. Exp. Criminol.* 7 27–56. 10.1007/s11292-010-9109-1

[B109] Tokunaga R. S. (2010). Following you home from school: a critical review and synthesis of research on cyberbullying victimization. *Comp. Hum. Behav.* 26 277–287. 10.1016/j.chb.2009.11.014

[B110] Twyman K. Saylor C. Taylor L. A. Comeaux C. (2010). Comparing children and adolescents engaged in cyberbullying to matched peers. *Cyberpsychol. Behav. Soc. Netw.* 13 195–199. 10.1089/cyber.2009.0137 20528278

[B111] Walrave M. Heirman W. (2011). Cyberbullying: predicting victimization and perpetration. *Child. Soc.* 25 59–72. 10.1111/j.1099-0860.2009.00260.x

[B112] Watson D. Clark L. A. Tellegen A. (1988). Development and validation of brief measures of positive and negative affect: the PANAS scales. *J. Pers. Soc. Psychol.* 54 1063–1070. 10.1037/0022-3514.54.6.1063 3397865

[B113] Williford A. Elledge L. C. Boulton A. J. DePaolis K. J. Little T. D. Salmivalli C. (2013). Effects of the KiVa antibullying program on cyberbullying and cybervictimization frequency among Finnish youth. *J. Clin. Child Adolesc. Psychol.* 42 820–833. 10.1080/15374416.2013.787623 23659182

[B114] Wright M. F. Yanagida T. Aoyama I. Dědková L. Li Z. Kamble S. V. (2016). Differences in coping strategies for public and private face-to-face and cyber victimization among adolescents in six countries. *Int. J. Dev. Sci.* 10 43–53. 10.1177/0022022116675413

[B115] Ybarra M. L. Diener-West M. Leaf P. J. (2007). Examining the overlap in internet harassment and school bullying: implications for school intervention. *J. Adolesc. Health* 41 42–50. 10.1016/j.jadohealth.2007.09.004 18047944

[B116] Ybarra M. L. Mitchell K. J. (2004). Online aggressors/targets, aggressors, and targets: a comparison of associated youth characteristics. *J. Child Psychol. Psychiatry* 45 1308–1316. 10.1111/j.1469-7610.2004.00328.x 15335350

[B117] Ybarra M. L. Mitchell K. J. (2007). Prevalence and frequency of Internet harassment instigation: implications for adolescent health. *J. Adolesc. Health* 41 189–195. 10.1016/j.jadohealth.2007.03.005 17659224

[B118] Zahn-Waxler C. Robinson J. L. Emde R. N. (1992). The development of empathy in twins. *Dev. Psychol.* 28 1038–1047. 10.1037/0012-1649.28.6.1038

[B119] Zych I. Ortega-Ruiz R. DelRey R. (2015). Systematic review of theoretical studies on bullying and cyberbullying: facts, knowledge, prevention and intervention. *Aggress. Violent Behav.* 23 1–21. 10.1016/j.avb.2015.10.001

